# Hydroxychloroquine Retinopathy in a UK Single-Centre Cohort: A Retrospective Study of Risk Factors and Dosing Trends

**DOI:** 10.7759/cureus.99096

**Published:** 2025-12-13

**Authors:** Michael Milad, Catharine Kwok, Oliver Wong, Marcela Bohn

**Affiliations:** 1 Ophthalmology, West Hertfordshire NHS Trust, Watford, GBR; 2 Ophthalmology, Moorfields Eye Hospital, London, GBR

**Keywords:** hydroxychloroquine retinopathy, ophthalmic research, renal impairment, retinal toxicity, tamoxifen ocular toxicity

## Abstract

Background

Hydroxychloroquine (HCQ) is a widely prescribed medication for autoimmune and inflammatory conditions. However, long-term use carries a risk of retinal toxicity. The prevalence of hydroxychloroquine retinopathy is higher than initially thought, and several clinical and pharmacological factors have been implicated as contributors. By optimising its prescription and monitoring, we can minimise preventable visual morbidity.

Objective

This study aimed to evaluate the prevalence of hydroxychloroquine retinopathy within a UK monitoring cohort and assess the impact of recognised risk factors, including daily dose relative to body weight, treatment duration, renal function and concurrent tamoxifen use.

Methods

A retrospective study was conducted at Watford General Hospital, West Hertfordshire NHS Trust, as part of an audit of clinical care. Patients reviewed in the dedicated hydroxychloroquine monitoring clinic between October 2023 and December 2024 were included. Demographic and clinical data were extracted, including age, sex, indication, dose (mg/kg/day), treatment duration, renal function, tamoxifen use, ocular history and evidence of toxicity. Subgroup analyses were carried out. Ethical approval was waived as the study was registered as an audit and was retrospective in nature.

Results

Data completeness was generally high, with six out of eight data items having reporting of over 90% of the cohort. However, treatment indication and precise dosing data were underreported in some records. A notable proportion of patients exceeded the recommended 5 mg/kg/day dosing threshold. Renal impairment and tamoxifen use were infrequent and not significantly associated with increased risk in this cohort. Subgroup analysis demonstrated that patients with toxicity were older, had been treated for a longer duration and received higher weight-adjusted doses compared to unaffected patients.

Conclusion

Hydroxychloroquine retinopathy was observed in association with higher doses and longer treatment duration, consistent with international literature. The enhanced reporting of dose and treatment indication, alongside stringent adherence to dosing guidelines, will improve patient safety. These findings highlight the importance of early monitoring and dose optimisation in reducing the risk of irreversible toxicity.

## Introduction

Hydroxychloroquine (HCQ), originally developed as an antimalarial drug, is widely used for autoimmune conditions such as systemic lupus erythematosus and rheumatoid arthritis, due to its favourable safety profile [1-4]. However, long-term use carries the risk of retinal toxicity, which can cause irreversible vision loss if undetected [5].

Contemporary studies using modern imaging techniques have raised the estimated prevalence of HCQ retinopathy, with rates approaching 7.5% amongst long-term users [6,7]. This has led both the American Academy of Ophthalmology and the Royal College of Ophthalmologists (RCOphth) to update their screening recommendations [8,9]. They have highlighted the need for weight-based dosing, the avoidance of daily doses exceeding 5 mg/kg of actual body weight and annual multimodal screening after five years of treatment or earlier in high-risk groups.

There have been multiple risk factors for toxicity that have been identified, including higher daily dose, cumulative duration of use, renal impairment, tamoxifen exposure and older age. Despite clear guidance, real-world prescribing practices frequently diverge from recommended dosing thresholds, and a significant proportion of patients continue to receive higher-than-advised daily doses [10,11]. By understanding how these risks manifest in local clinical populations, we can improve prescribing safety and screening efficiency.

Within the United Kingdom, the expansion of dedicated HCQ monitoring services provides an opportunity to assess compliance with guideline-recommended dosing and screening pathways and to evaluate the prevalence of retinopathy in practice [12]. However, there remains limited UK-based evidence describing the distribution of risk factors and the real-world burden of toxicity in monitored cohorts.

This study therefore aimed to determine the prevalence of HCQ retinopathy within a specialist UK monitoring clinic and examine how recognised risk factors relate to toxicity in this population. By characterising dosing patterns and identifying areas for improved documentation and risk assessment, our findings seek to inform safer prescribing and earlier detection strategies.

## Materials and methods

Study design and setting

We performed a retrospective cohort study at Watford General Hospital, West Hertfordshire NHS Trust. All patients attending the dedicated hydroxychloroquine monitoring clinic between October 2023 and December 2024 were considered for inclusion. Given the retrospective nature of the study and the use of anonymised clinical data, as well as audit registration and approval by the audit lead, institutional review determined that ethical approval was waived.

Screening workflow

Patients referred for HCQ monitoring underwent multimodal imaging, in accordance with the Royal College of Ophthalmologists' recommendations. Standard investigations included spectral-domain optical coherence tomography (SD-OCT) and fundus autofluorescence (FAF). Images were reviewed in a consultant-led virtual clinic. If there was suggested toxicity, the patient could order additional tests such as 10-2 visual fields and electrodiagnostics or to see them face to face.

Study population

Patients were eligible if they were current or past HCQ users, attended at least one monitoring visit during the study period and had sufficient information available to calculate the daily weight-adjusted dose and determine retinal status. Patients were excluded if clinical documentation did not allow the calculation of dose in mg/kg, if no retinal imaging or assessment of retinopathy was available or if HCQ had been discontinued before screening without any ophthalmic evaluation.

Data collection and variables

Data were extracted from the electronic patient record system by three independent reviewers (MM, CK and OW). Where discrepancies arose, a meeting between the reviewers was organised to discuss concerns or discrepancies. Risk factors recorded were renal impairment, concurrent or previous tamoxifen use and any documented ocular comorbidities. The primary outcome was the presence of HCQ retinopathy, with toxicity demonstrated on at least two modalities, typically SD-OCT, FAF and 10-2 visual field testing.

Definitions

High-dose HCQ therapy was defined as a daily dose exceeding 5 mg/kg of actual body weight. Renal impairment was defined as an estimated glomerular filtration rate (eGFR) of less than 60 mL/minute/1.73 m². Retinopathy referred to multimodal imaging features consistent with HCQ toxicity.

Measures to reduce bias

To minimise bias, the definitions of variables were standardised before extraction. Any ambiguities were discussed in consensus meetings. Definitions of terms were standardised before extraction. Where dosing information was incomplete, we calculated variables using weight measurements where possible.

Statistical analysis

All statistical analyses were conducted using Microsoft Excel (Microsoft Corporation, Redmond, WA). Continuous variables were expressed as mean ± standard deviation (SD) if normally distributed. Categorical variables were reported as counts and percentages. Patients with and without HCQ retinopathy were compared. Two-tailed, independent sample t tests were applied for continuous data. Chi-square tests were used to compare categorical variables. Statistical analyses were carried out using Microsoft Excel, and results were independently cross-checked to minimise error. We explored the associations between retinopathy and high-dose therapy; treatment duration, renal impairment, tamoxifen use and age were explored. A p-value of <0.05 was considered statistically significant, with values of <0.001 marked on figures as very significant.

## Results

Cohort overview

**Figure 1:**
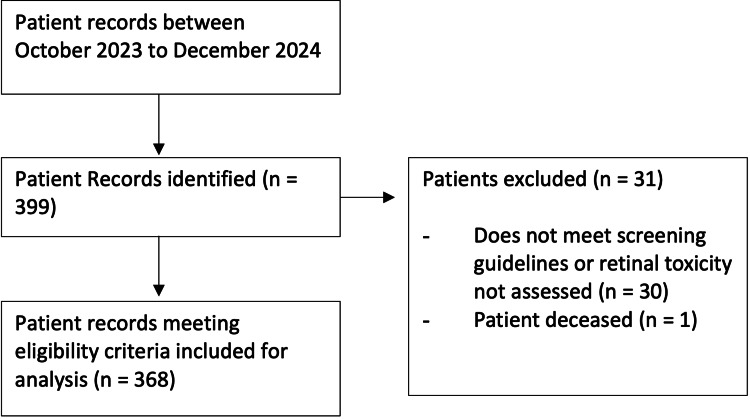
Flow chart summarising included and excluded studies.

During the study period, 399 patients attended the hydroxychloroquine monitoring clinic. After excluding 31 patients for incomplete data, 368 patients were included in the final analysis (Figure 1).

Data reporting

Data reporting was generally high, with demographic and treatment duration well documented (Table 1). However, the indication for treatment was missing in 16.8% of cases, and the exact daily dose could not be confirmed in 14.7%. These gaps may reflect variability in how prescribing information is transferred from rheumatology to ophthalmology records.

**Table 1:** A summary of data reporting in the cohort of 368 patients.

Data Item	Number of Patients Reported (N = 368)	Proportion of Cohort (%)
Toxicity	368	100
Treatment dose (daily or per kilogram)	314	85.3
Duration of treatment	359	97.5
Patient weight	342	92.9
Treatment indication	310	84.2
Kidney function	366	99.4
Tamoxifen use	367	99.7
Ocular history	368	100

Baseline characteristics

The cohort was predominantly women (88.8%), with a mean age of 60.3 years (SD: 13.3). The most common indications for HCQ therapy were rheumatoid arthritis (56.4%) and systemic lupus erythematosus (16.1%). The mean treatment duration was 72.4 years (SD: 17.6). The mean daily dose was 3.88 mg/kg/day (SD: 1.5). Table 2 summarises baseline demographic and clinical characteristics.

**Table 2:** A summary of baseline characteristics in the cohort of 368 patients. HCQ, hydroxychloroquine; RA, rheumatoid arthritis

Characteristic	Number (Standard Deviation {SD})
Total number of patients	368
Mean age (years)	60.3 (SD: 13.3)
Proportion of women (%)	88.2
RA as indication for HCQ, n (percentage of reported)	175 (56.5)
Mean dose (mg/kg)	3.88 (SD: 1.5)
Mean duration of treatment (years)	9.19 (SD: 5.34)
Mean weight (kg)	72.4 (SD: 17.6)

Prevalence of retinopathy and risk factor prevalence

Signs of hydroxychloroquine retinopathy were present in 14 patients (3.8%) of the cohort. Seventy-seven patients (24.4%) were taking a daily dose above the recommended 5 mg/kg/day. Sixteen patients had renal dysfunction, five had previously used tamoxifen and one patient had both renal dysfunction and previous tamoxifen use.

Subgroup analyses

**Figure 2:**
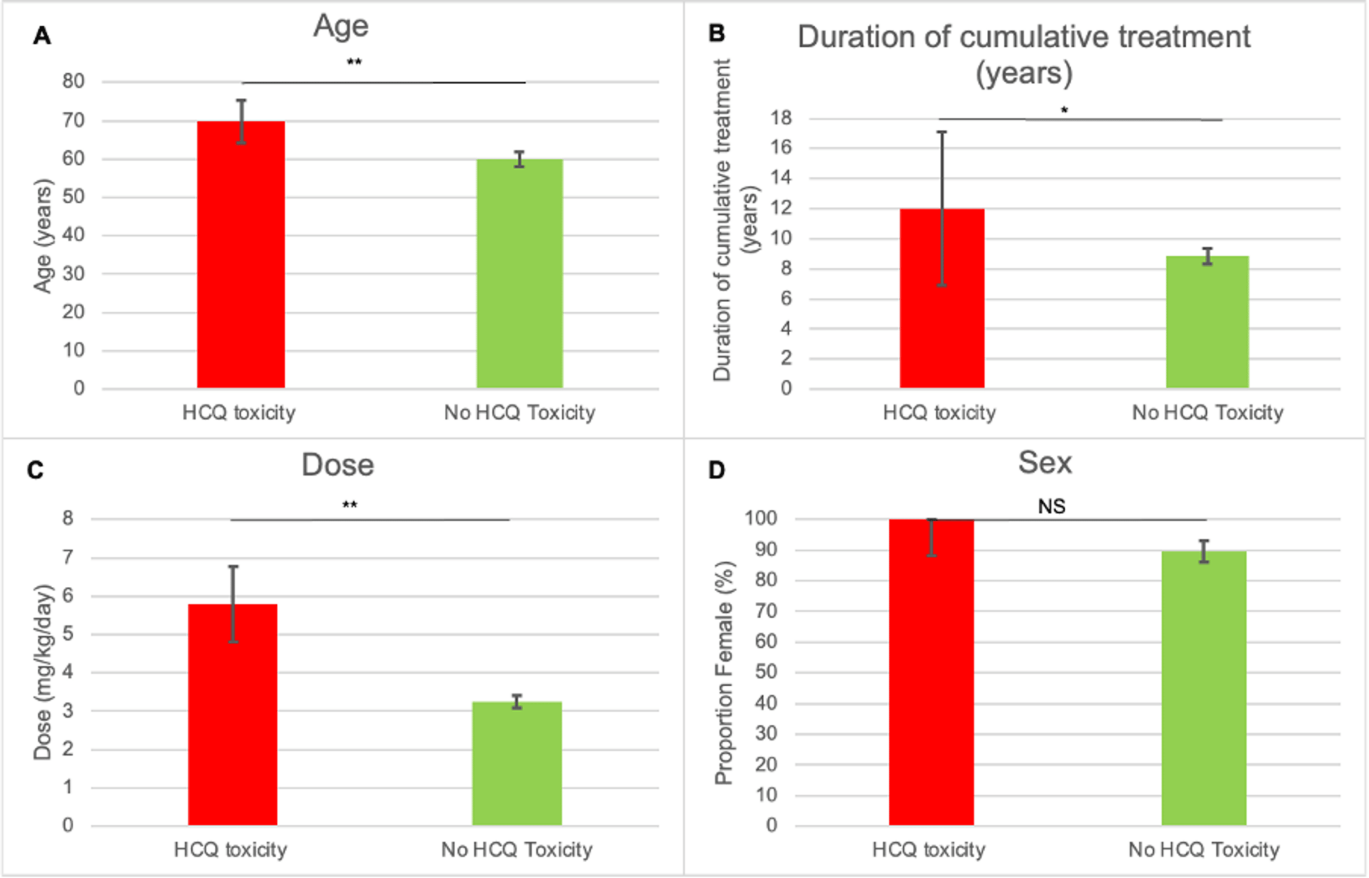
A comparison between the HCQ and non-HCQ cohorts. Age (A), duration of cumulative treatment (B), dose (C) and sex (D) are compared between both groups. Bars indicate confidence interval. *P < 0.05 **P < 0.001 NS, non-significant difference; HCQ, hydroxychloroquine

**Figure 3:**
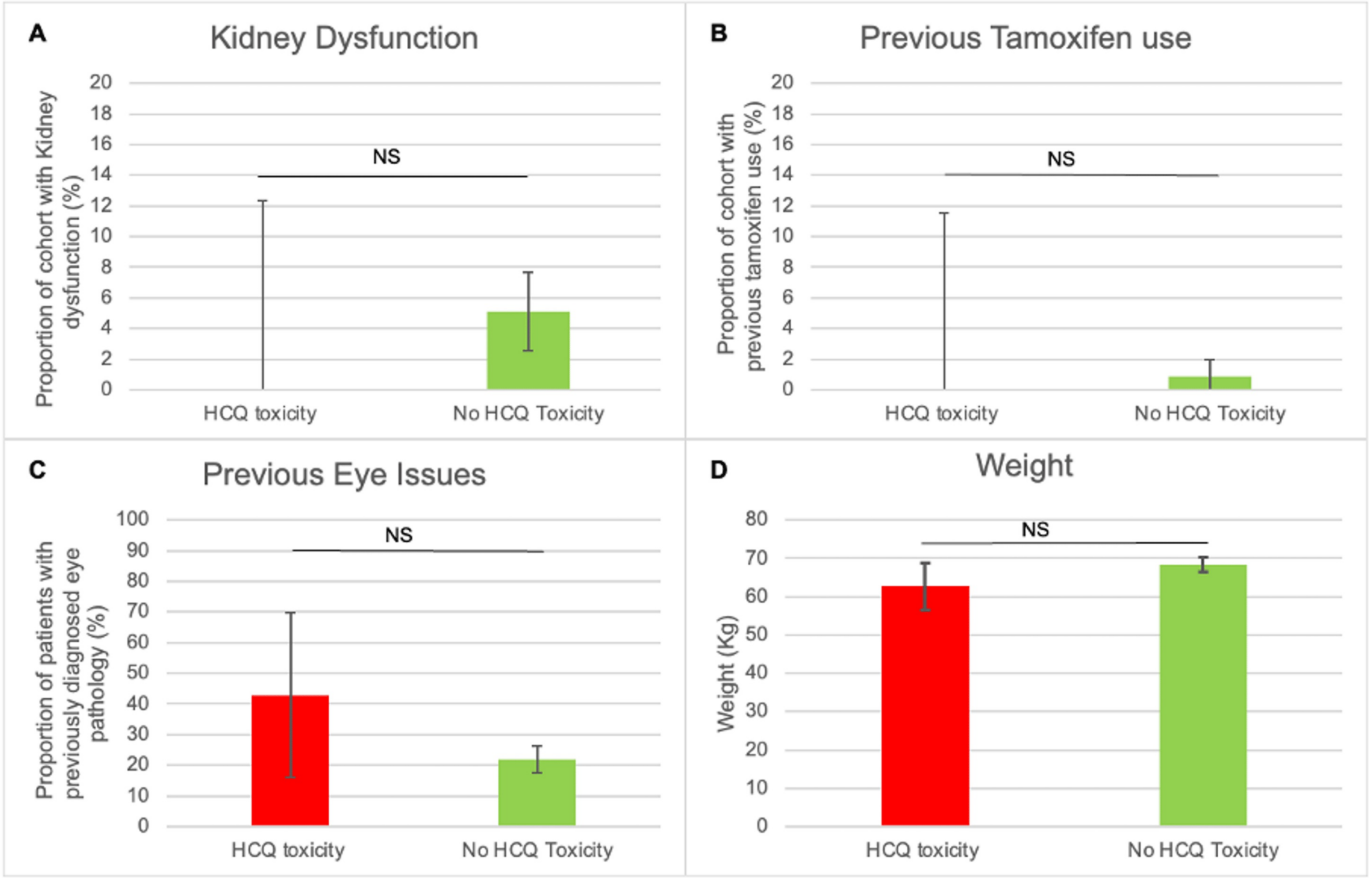
A comparison between the HCQ and non-HCQ cohorts. The presence of kidney dysfunction (A), previous tamoxifen use (B), previous ocular issues (C) and weight (D) are compared between both groups. NS, non-significant difference; HCQ, hydroxychloroquine

Compared to unaffected patients, those with toxicity were exposed to a higher dose, treated for longer and older. There was no difference between both groups' mean weight, sex, presence of renal dysfunction, previous tamoxifen use or previous ocular history (Figure 2 and Figure 3).

## Discussion

This retrospective analysis of 368 patients attending a dedicated hydroxychloroquine monitoring clinic at a UK district general hospital provides important insights into prescribing patterns, risk factor prevalence and the occurrence of retinopathy. The prevalence of hydroxychloroquine retinopathy in our cohort was 3.8%, which is lower than the 7.5% prevalence reported in the large Kaiser Permanente cohort from the United States but consistent with smaller studies that have reported prevalence rates ranging from 1% to 5% depending on case definition and screening methods [13-15]. The discrepancy in prevalence may be attributable to shorter mean duration of exposure in our population, differences in dosing patterns or the relatively small number of patients with renal impairment and tamoxifen use in this cohort.

Our findings reinforce the role of daily dose and duration of therapy as the strongest predictors of toxicity. Patients with retinopathy were significantly older, had received higher weight-adjusted daily doses and had been treated for longer durations than those without toxicity. These associations align with prior studies, which consistently demonstrate that risk increases steeply after five years of therapy and is markedly higher in patients exceeding the recommended 5 mg/kg/day threshold [16,17]. Importantly, almost one-quarter of patients in our cohort were prescribed doses above this threshold, indicating that deviations from best practice remain common in real-world settings. Addressing this issue is critical, as dose reduction is a modifiable factor that can substantially reduce long-term risk.

Renal impairment and tamoxifen use were uncommon in this population and did not show a statistically significant association with retinopathy. This is in contrast with larger studies that have identified both as significant risk enhancers [1,7]. The lack of association in our data likely reflects limited statistical power due to small numbers rather than an absence of biological effect. Clinicians should therefore continue to consider renal function and concomitant tamoxifen therapy when assessing risk.

From a service delivery perspective, our study highlights both strengths and weaknesses in data capture within a dedicated monitoring clinic. Reporting of demographic and treatment duration was excellent, with over 97% completeness. However, indications for treatment and precise daily dose calculations were less reliably documented, missing in approximately 15% of cases. Improved communication between prescribing specialties and ophthalmology, including systematic transfer of prescription details, would strengthen data quality and enhance patient safety [18].

The findings of the study also highlight the importance of continued education for prescribing clinicians regarding HCQ dosing and toxicity. Many patients were prescribed a dose exceeding the recommended 5 mg/kg/day threshold, often due to reliance rather than actual body weight. Hence, reinforcing education on weight-based dosing across rheumatology, dermatology and general practice is essential. The early identification of at-risk patients and proactive dose review at initiation can prevent avoidable toxicity whilst ensuring those at a higher risk undergo earlier and more frequent monitoring.

The strengths of this study include its relatively large sample size for a single-centre UK study, the use of multimodal imaging in line with RCOphth recommendations and systematic subgroup analyses of key risk factors. Limitations include its retrospective design, reliance on routinely collected data and the potential underestimation of toxicity in patients with incomplete follow-up. The relatively short mean treatment duration also limits direct comparison with long-term cohorts where prevalence is higher. Finally, as a single-centre study, findings may not be generalisable to all UK populations.

Nevertheless, these results carry important clinical implications. First, they underscore the need for strict adherence to guideline-recommended dosing, with attention to weight-adjusted calculations. Second, they support the ongoing expansion of dedicated monitoring clinics across the NHS, which allow the systematic detection of retinopathy at earlier, presymptomatic stages. Third, they highlight the importance of improving the documentation of prescribing details, which is crucial for both patient safety and research.

In light of the study's findings, a more standardised baseline assessment at the start of HCQ therapy can help improve the early detection of toxicity. By recording markers such as visual acuity and SD-OCT at initiation, we can have a baseline to compare future scans against. This would be particularly useful for patients with multiple risk factors, hence putting them at a significantly increased risk of developing toxicity. Furthermore, early abnormalities on multifocal electroretinography (mfERG) can precede obvious structural changes and provide additional value. Notably, recent work using artificial intelligence to analyse mfERG waveforms has shown encouraging results in predicting early HCQ-related retinal toxicity [19]. These developments highlight the potential for mfERG to contribute to future risk-stratification strategies, and prospective studies are needed to explore how it could be incorporated effectively into long-term monitoring pathways.

Future research should focus on longer-term follow-up of UK cohorts, the exploration of cumulative dose thresholds in different risk groups and the evaluation of the cost-effectiveness of current screening intervals. Prospective studies could also clarify the interaction between renal impairment, tamoxifen use and other comorbidities in modulating toxicity risk.

## Conclusions

In summary, this retrospective cohort study identified a 3.8% prevalence of hydroxychloroquine retinopathy in a monitored UK population. Toxicity was strongly associated with higher daily doses and longer duration of treatment, consistent with international evidence. Renal impairment and tamoxifen use were uncommon and not significantly associated with toxicity in this cohort, though these remain recognised risk factors in the wider literature.

Our findings highlight the ongoing challenge of overdosing relative to body weight, with nearly one-quarter of patients exceeding the recommended 5 mg/kg/day threshold. Improved adherence to prescribing guidelines and the systematic communication of treatment details are essential to minimise risk. Dedicated monitoring clinics play a vital role in identifying early toxicity and safeguarding vision; however, further work is needed to optimise both prescribing and screening practices.
